# Overexpression of TREM2 enhances glioma cell proliferation and invasion: a therapeutic target in human glioma

**DOI:** 10.18632/oncotarget.6221

**Published:** 2015-10-24

**Authors:** Xiao-Qiang Wang, Bang-Bao Tao, Bin Li, Xu-Hui Wang, Wen-Chuan Zhang, Liang Wan, Xu-Ming Hua, Shi-Ting Li

**Affiliations:** ^1^ Department of Neurosurgery, Xinhua Hospital, Shanghai Jiaotong University School of Medicine, Shanghai, China

**Keywords:** TREM2, glioma, proliferation, invasion, chemokine pathway

## Abstract

Gliomas are the most common and aggressive type of primary adult brain tumors. Although TREM2 mutation is reported to be related to Nasu-Hakola disease and Alzheimer's disease, little is known about the association between TREM2 and gliomas. Here, we reported that TREM2 was significantly overexpressed in glioma tissues compared with non-tumorous brain tissues. Furthermore, TREM2 expression was closely related to pathological grade and overall survival of patients with gliomas. Down-regulation of TREM2 in two glioma cell lines, U87 and U373, resulted in a significant reduction in cell proliferation, migration and invasion and a dramatic increase in S phase arrest and apoptosis. *In vivo* tumorigenesis experiment also revealed that depletion of TREM2 expression inhibited U87 cell proliferation. Moreover, based on gene set enrichment analysis (GSEA) with The Cancer Genome Atlas (TCGA) dataset, we found that TREM2 was positive related to Kyoto Encyclopedia of Genes and Genomes (KEGG) apoptosis, Cromer metastasis and KEGG chemokine pathways, which was further validated by western blot in TREM2 knockdown glioma cells and indicated a possible mechanism underlying its effects on glioma. In summary, our study suggests that TREM2 may work as an oncogene and a new effective therapeutic target for glioma treatment.

## INTRODUCTION

Gliomas are the most common primary brain tumor, accounting for 45% of all primary brain tumors [1, 2]. According to the 2007 World Health Organization (WHO) classification, gliomas are divided into four pathological types (Grade I, II, III, and IV), of which glioblastoma multiforme (GBM, WHO grade IV) is the most frequent and aggressive malignant type [2]. Despite current advances in diagnostic methods and therapeutic strategies, the prognosis of GBM remains poor, with an average survival time of less than one year [3]. Consequently, a better understanding of the molecular mechanisms underlying the formation and development of gliomas will help identify novel therapeutic targets and develop strategies for the treatment of gliomas.

The triggering receptors expressed on myeloid cells 2 (TREM2) belong to a family of transmembrane receptors expressed on myeloid cells [4]. TREM2 is composed of a variant-type extracellular domain, a charged transmembrane domain and a short cytoplasmic tail. TREM2 associates with the adaptor protein DAP12 [4], which contains an immunoreceptor tyrosine-based activation motif (ITAM). DAP12 can recruit Syk and Zap70, and activates PI3K, phospholipase C, and Vav signaling cascades [5-7]. TREM2 mainly functions on dendritic cells (DCs), osteoclasts and microglia. Mutations of TREM2 cause skeletal and psychotic abnormalities called Nasu-Hakola disease (NHD) [8], or a neurodegenerative disorder characterized by numerous deficits in neurotransmitter function called Alzheimer's disease [9]. The mutation of TREM2 in these two diseases indicates its function in bone remodeling and the central nervous system. Recently, several studies have reported that TREM1, another member of the TREM family, exerted tumor promoting effects on human malignancies including lung cancer [10, 11], colon cancer [12] and hepatocellular carcinoma (HCC) [13-15]. However, few investigations have been conducted on the association between TREM2 and human malignancies. Here, considering the association between TREM2 and central nervous system disease, we sought to study the role of TREM2 in glioma development.

In the present study, we showed that up-regulation of TREM2 in human gliomas is closely related to tumor progression, and knockdown of TREM2 can inhibit the proliferation, adhesion, migration and invasion of glioma cells. Furthermore, gene set enrichment analysis (GSEA) using The Cancer Genome Atlas (TCGA) dataset showed that TREM2 was positive related with Kyoto Encyclopedia of Genes and Genomes (KEGG) apoptosis, Cromer metastasis and KEGG chemokine pathways, which was further validated in glioma cells with TREM2 silenced. Our data provide new insights into the molecular function of TREM2 as well as its regulatory mechanisms in gliomas.

## RESULTS

### TREM2 Upregulation in human glioma tissues

**Figure 1:**
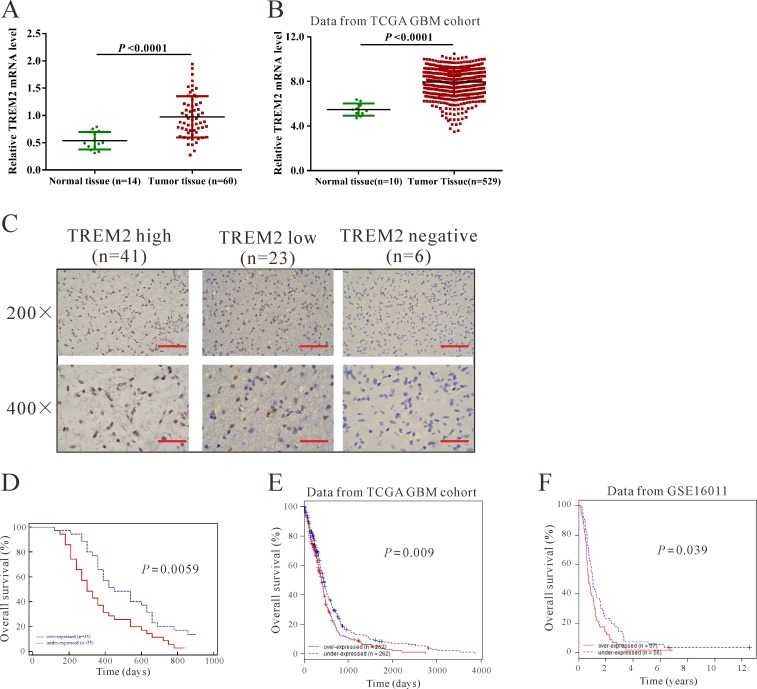
TREM2 was overexpressed in glioma tissues **A.** TREM2 mRNA level was significantly higher in glioma tissues (*n* = 60) than in non-tumorous brain tissues (*n* = 14) from patients admitted to Xinhua Hospital from January 2009 to December 2010 (*P* < 0.0001). **B.** TREM2 expression was significantly increased in glioma tissues (*n* = 529) compared with normal tissues of patients (*n* = 10) from the TCGA GBM dataset (*P* < 0.0001). **C.** Expression of TREM2 was determined by immunohistochemistry staining in glioma tissues. Low power (200×) scale bars: 100 μm, high power (400×) scale bars: 50 μm. **D.** The overall survival time of 70 patients with glioma (*P* < 0.001). **E.** Survival analysis of patients from TCGA GBM dataset (*P* < 0.01). **F.** Survival analysis of patients from GSE16011 dataset (*P* < 0.05).

We first tested the mRNA levels of TREM2 in 60 snap-frozen glioma tissues and 14 normal brain tissues using real-time quantitative PCR (RT-qPCR). Compared with normal brain tissues, significant upregulation of TREM2 was observed in glioma tissues (Figure 1A, *P* < 0.0001). Then, we re-analyzed high throughput RNA-sequencing data of the GBM cohort of The Cancer Genome Atlas (TCGA, https://tcga-data.nci.nih.gov/tcga/tcgaCancerDetails.jsp?diseaseType=GBM&diseaseName=Glioblastoma%20multiforme) and found that TREM2 expression was significantly increased in glioma tissues compared with normal brain tissues (Figure 1B, *P* < 0.001).

To assess the protein levels of TREM2 in glioma tissues, immunohistochemistry (IHC) staining of TREM2 was performed in 70 human glioma specimens. High expression (more than 25% of positive-stained tumor cells), low expression (less than 25% of positive-stained tumor cells) and non-expression of TREM2 was observed in 41, 23 and 6 cases of glioma, respectively (Figure 1C).

### Upregulation of TREM2 is associated with the progression of gliomas

According to IHC staining results, all 70 glioma tissue samples were divided into two groups. Group 1 was the high TREM2 expression group, and Group 2 was the low and negative TREM2 expression group. Then, the association between TREM2 expression and various clinicopathological parameters of glioma tissues was analyzed, as shown in Table 1. Chi-square test showed that the increased expression of TREM2 was significantly associated with pathological grade (P < 0.01). However, there was no significant association between TREM2 expression and other clinicopathological parameters, including patients' gender and age at diagnosis and tumor size (Table 1).

**Table 1:** Relationship between TREM2 expression and different clinicopathological features in human glioma patients (*n* = 70)

Parameters	Characteristic	TREM2		*P*-value
		High (*n* = 41)	Low and Negative (*n* = 29)	
Age (years)	>=55	25	15	0.4717
	<55	16	14	
Gender	Male	14	12	0.6188
	Female	27	17	
Tumor size	>=4.5 cm	24	13	0.3327
	<4.5 cm	17	16	
WHO grade	I/II	12	18	0.0079>**
	III/IV	29	11	

WHO, World Health Organization.

Note: *P* values are from chi-square test and were significant at <0.05.

** *P* < 0.01.

Furthermore, the association between TREM2 expression and prognosis in patients with gliomas was determined by analyzing our own data, as well as the TCGA GBM and GSE 16011 datasets [16] (http://www.ebi.ac.uk/arrayexpress/experiments/E-GEOD-16011/?query=GSE16011). According to the log-rank test and Kaplan-Meier analysis, the expression level of TREM2 in gliomas displayed a significant correlation with the patients' survival time (Figure 1D-1F, *P* < 0.05).

### Knockdown TREM2 expression inhibits growth of glioma cells *in vitro*

**Figure 2:**
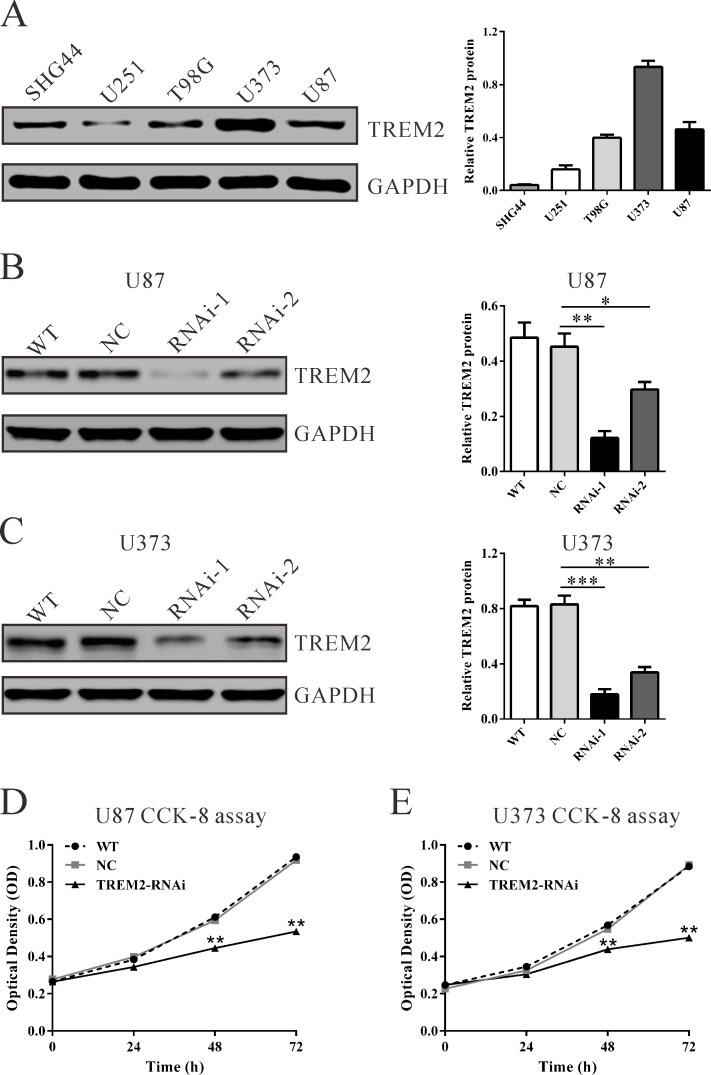
Suppressing TREM2 expression inhibited cell growth of glioma cells **A.** TREM2 expression level in five glioma cell lines was analyzed by western blot. GAPDH was also detected as the internal control. Representative western blots (left panel) and quantitative results were shown (right panel). **B.**, **C.** Expression of TREM2 in U87 and U373 cells was analyzed by western blot. **D.**, **E.** Cell proliferation was detected in siRNA treated and untreated U87 and U373 cells by CCK-8 assay. WT: wild type cells; NC: scrambled siRNA transfected cells; RNAi-1 and RNAi-2: TREM2-siRNA-1 and -2 transfected cells. Data were based on at least 3 independent experiments, and shown as the mean ± S.D. (*P < 0.05, **P < 0.01, ***P < 0.001 compared with NC).

To further explore its biological role in gliomas, we knockdown the expression of TREM2 in U87 and U373 cells, which expressed high levels of TREM2 by siRNA transfection. As shown in Figure 2, two TREM2 siRNAs (TREM2-siRNA-1 and TREM2-siRNA-2) were able to efficiently suppress endogenous TREM2 expression in both glioma cells, whereas TREM2 expression remained unaffected in control siRNA-transfected cells (NC). TREM2-siRNA-1 showed higher knockdown efficiency than TREM2-siRNA-2 and was chosen for the following assays. The suppression ratio of TREM2 protein expression by TREM2-siRNA-1 in U87 and U373 was 73.0% and 82.4%, respectively.

Subsequently, glioma cell proliferation was detected *in vitro*. Depletion of TREM2 resulted in a significant decrease at 24 h, 48 h and 72 h in viability compared with the NC group in both U87 and U373 cells in a CCK-8 assay (Figure 2, *P* < 0.01). These results indicated an anti-proliferation role of TREM2-siRNA in glioma cells.

### Depletion of TREM2 induces S-phase arrest and apoptosis of glioma cells

**Figure 3:**
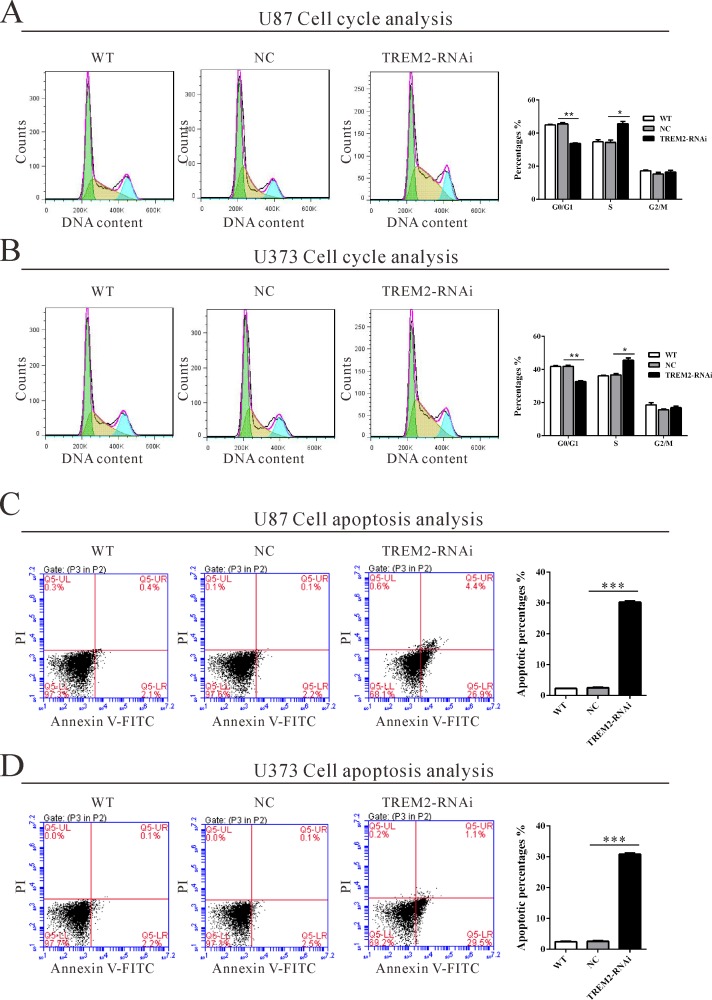
Suppressing TREM2 expression induced S phase arrest and apoptosis in glioma cells U87 and U373 cells were transfected with indicated siRNA and collected 48 hours later cells. **A.**, **B.** Cell cycle profile was analyzed using flow cytometry. **C.**, **D.** Cell apoptosis was analyzed by Annexin V/PI staining. Data were based on at least 3 independent experiments, and shown as the mean ± S.D. (*P < 0.05, ** *P* < 0.01, ***P < 0.001 compared with NC).

To determine whether TREM2 influences the cell cycle of glioma cells, cell cycle distribution was assessed in TREM2 knockdown cells. Flow cytometry analysis revealed that the population of G0/G1 phase cells in U87 (Figure 3A) transfected with TREM2 siRNA was significantly decreased by 26.0% (**P < 0.01), and S phase cells increased by 33.0%, compared with NC and wild-type (WT) cells. Similar results were obtained in U373 cells (Figure 3B).

We then evaluated the apoptotic function of TREM2 in glioma cells by Annexin V-FITC/PI staining assay. As shown in Figure 3C and 3D, flow cytometry analysis revealed that knockdown of TREM2 in both glioma cells significantly induced cell apoptosis approximately 12-fold compared with corresponding control cells (NC).

### Down-regulation of TREM2 expression inhibits adhesive, invasive and migratory ability in glioma cells

**Figure 4:**
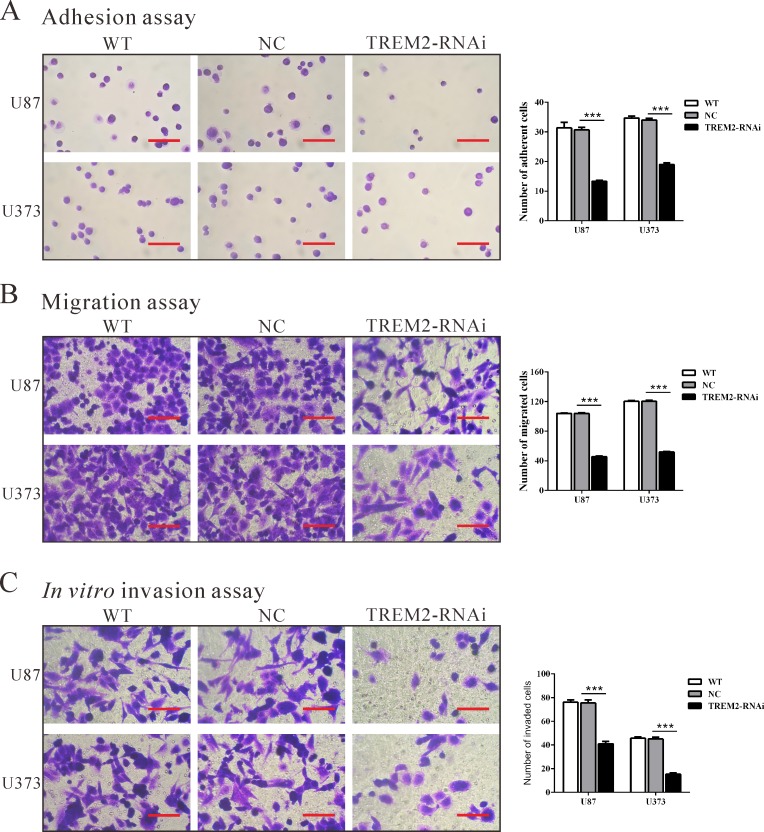
Silencing of TREM2 inhibited cell adhesion, migration and invasion in glioma cells U87 and U373 cells were transfected with siRNA control (NC) or TREM2-siRNA. Cell adhesion **A.**, migration **B.**, and invasion assay **C.** were performed. Representative images (left panel) and the quantification (right panel) are shown. Scale bars: 100 μm. Data were based on at least 3 independent experiments, and shown as the mean ± S.D. (***P < 0.001 compared with NC).

To examine the effect of TREM2 on cell adhesion to matrix, cell adhesion assay was performed in fibronectin-coated plates. As shown in Figure 4A, TREM2-siRNA treatment caused a significant decrease in the adhesive capacity of both glioma cells (*P* < 0.001).

To examine the effect of TREM2 on cell migration, TREM2-RNAi, WT and NC cells were cultured in a Boyden chamber. After 24 h of incubation, compared with the NC cells (104 ± 1 cells and 120 ± 1 cells), both TREM2-RNAi-U87 and TREM2-RNAi-U373 glioma cells showed significantly decreased migratory ability (46 ± 1 cells and 52 ± 1 cells, respectively, both *P* < 0.001; Figure 4B).

Using a matrigel-coated transwell chamber, we determined changes in cell invasion after 24 h of incubation. After 24 h of incubation, similar numbers of WT and NC-transfected cells invaded through the matrigel (U87: WT, 76 ± 2; NC, 75 ± 3; U373: WT, 46 ± 1; NC, 45 ± 2), whereas a strongly inhibited invasive ability was observed in TREM2 knockdown cells (U87: 41 ± 2; U373: 15 ± 1, *P* < 0.001; Figure 4C). Our results suggested a role of TREM2 in the promotion of glioma invasion.

### Silencing of TREM2 suppresses tumorigenesis of glioma cells *in vivo*

**Figure 5:**
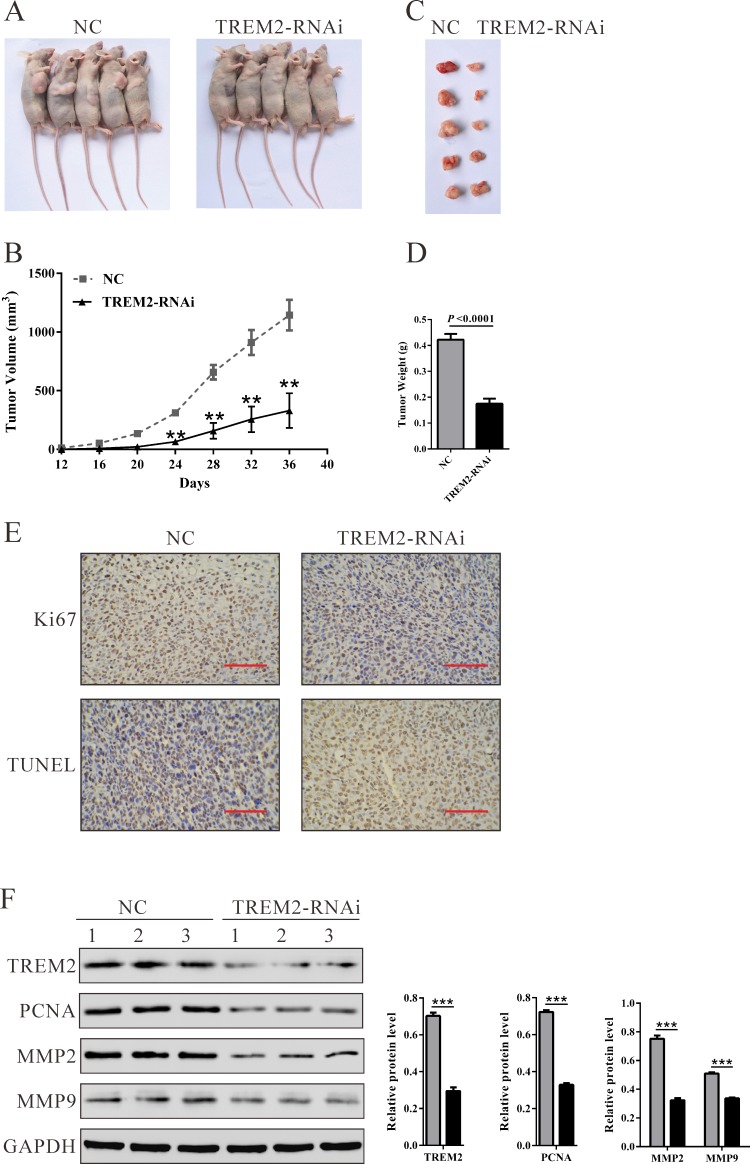
Knockdown of TREM2 in glioma cells reduced tumor growth *in vivo* **A.** U87 cells transfected with siRNA control (NC) or TREM2-siRNA were subcutaneously injected in athymic nude mice. Tumor growth was shown 36 days after injection. **B.** Time course analysis of tumor growth after injection (**P < 0.01 compared with NC). **C.** Mice were sacrificed and the tumors were isolated after 36 days. **D.** Tumor growth was significantly reduced in TREM2 knockdown tumors (*P* < 0.0001). **E.** Transplanted tumors with Ki67 immunostaining and TUNEL staining. **F.** The expression of TREM2, PCNA, MMP2 and MMP9 in xenograft from the nude mice was determined by western blot (**P < 0.01, ***P < 0.001 compared with NC).

To confirm the growth inhibitory effect of TREM2-siRNA *in vivo*, a xenograft tumor-bearing model was established by inoculating U87 cells into nude mice and treated with TREM-siRNA or control siRNA. As shown in Figure 5, TREM2-RNAi-treated tumors grew much slower than the control siRNA-treated tumors in mice. Mice in the TREM2-RNAi group and NC group were killed 36 days after inoculation, with average tumor weights of 0.17 ± 0.04 g and 0.42 ± 0.05 g, respectively (Figure 5D, *P* < 0.0001). These results demonstrated that TREM2-siRNA could exert a significant inhibitory effect on tumorigenesis of glioma cells *in vivo*.

Furthermore, compared with NC-ones, a significant decrease of Ki67-positive cells and a notable increase of apoptotic cells were observed in tumors formed from knockdown TREM2 cells by immunostaining and TUNEL assay (Figure 5E). Western blot analysis revealed that TREM2, proliferating cell nuclear antigen (PCNA) and invasion related proteins (MMP2 and MMP9) were also significantly decreased in tumors formed from knockdown TREM2 cells (Figure 5F).

### Identification of TREM2-associated biological pathways by gene set enrichment analysis (GSEA)

**Figure 6:**
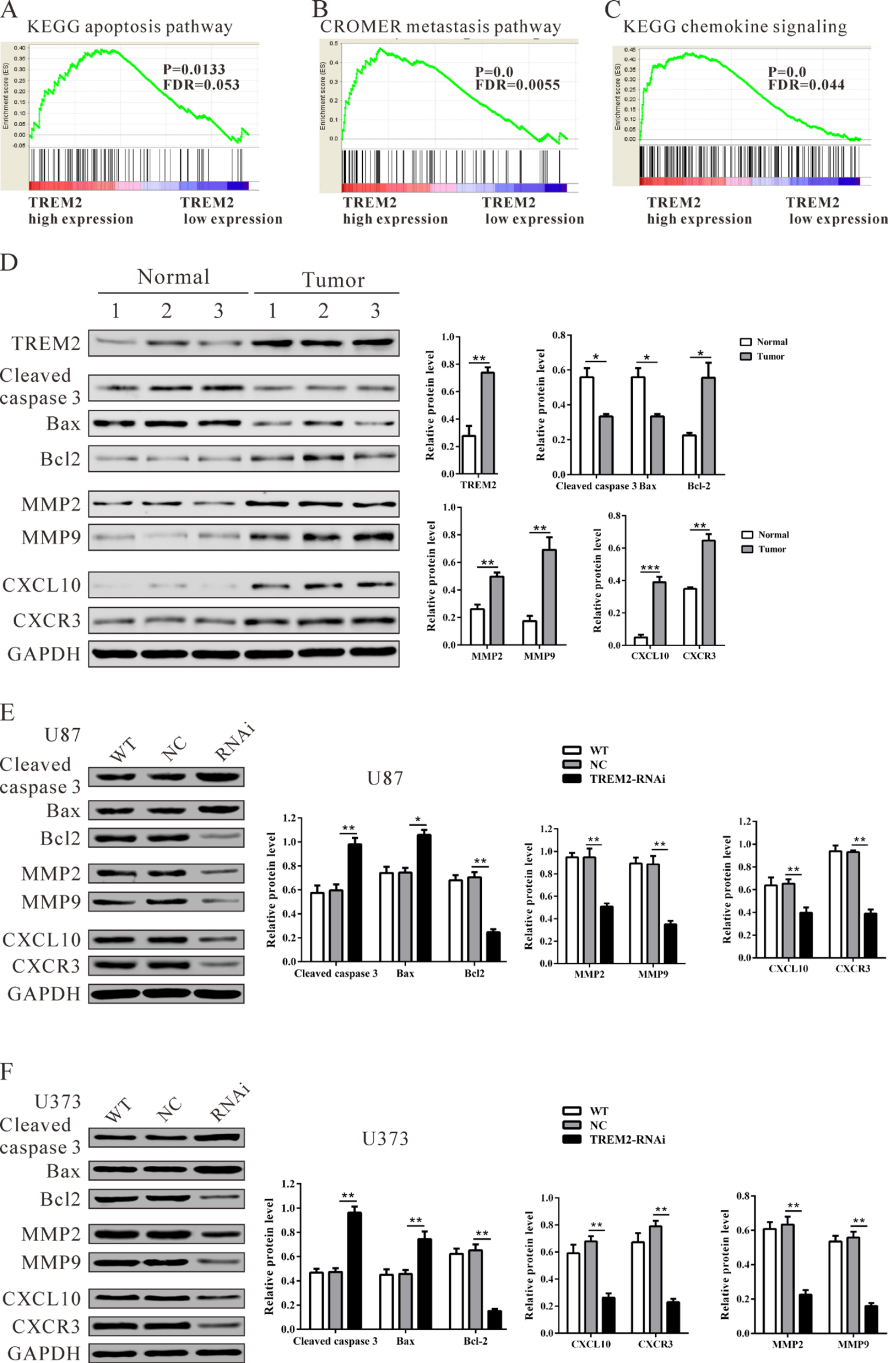
Mechanisms of TREM2 exert their functions in glioma cells Enrichment plots of gene expression signatures for KEGG apoptosis **A.**, Cromer invasion **B.** and KEGG chemokine pathways **C.** according to TREM2 expression levels. **D.**, **E.**, **F.** Protein levels of apoptosis-related factors (cleaved caspase 3 and Bad), anti-apoptosis (Bcl2), invasion (MMP2 and MMP9) and chemokine pathway related factors (CXCL10 and CXCR3) in glioma and normal tissues, and glioma cell lines (U87 and U373 cells) were detected by western blot. GAPDH was also detected as the control of sample loading. Representative western blots (left panel) and quantitative results were shown (right panel). Data were based on at least three independent experiments, and shown as the mean ± SD (*P < 0.05, ** *P* < 0.01, ***P < 0.001 compared with NC). WT: wild type cells; NC: scrambled siRNA transfected cells; RNAi: TREM2-siRNA transfected cells.

To assess the TREM2-associated pathways on an unbiased basis, we performed gene set analysis using data from the TCGA GBM cohort. GSEA was done using GSEA software version 2.0.1, obtained from the Broad Institute [17]. The expression level of TREM2 gene was used as the phenotype label. We used the nominal P value and NES to sort the pathways enriched in each phenotype and the pathways database as the gene sets in this study. KEGG apoptosis, Cromer metastasis and KEGG chemokine pathways were identified to be significantly associated with TREM2 expression in the TCGA BGM cohort (Figure 6A-6C).

### Validation of GSEA analysis of TREM2 in glioma cells

To validate the GSEA analysis of TREM2, we analyzed the protein levels of the above three pathways, related factors in glioma tissues, normal tissues, as well as TREM2-siRNA treated U87 and U373 cells. As shown in Figure 6D, the protein levels of TREM2, anti-apoptosis (Bcl2), invasion (MMP2 and MMP9) and chemokine pathway related factors (CXCL10 and CXCR3) were significantly higher in glioma tissues than in normal tissues, while the expression of apoptosis-related factors (cleaved caspase 3 and Bad) was remarkably lower in glioma tissues. Consistent with functional characterization *in vitro*, both TREM2 knockdown cell lines display an increase in apoptosis-related factors, but a decrease in anti-apoptosis, invasion and chemokine pathway related factors (Figure 6E and 6F).

## DISCUSSION

It is well known that mutation of TREM2 leads to NHD [8] and Alzheimer's disease [9], yet its association with gliomas has not been clarified. In the present study, we found that TREM2 expression was increased in glioma tissues compared with normal brain tissues, which was supported by glioma patients' data from TCGA. Moreover, TREM2 expression was associated with pathological grade and patients' survival rate (Figure 1 and Table 1). These data indicated the prognostic value of TREM2 in gliomas.

Gliomas are well-known not only for rapid proliferation but also for invading the surrounding brain tissue, resulting in an extremely poor prognosis. Our data affirmed that knockdown of TREM2 remarkably inhibited growth (Figure 2 and Figure 3), migration and invasion of glioma cells (Figure 4), while ectopic expression of TREM2 showed a lower expression glioma cell line, SHG44, promoted cell proliferation, migration and invasion (Figure S1). These results were consistent with the functions of TREM1 on HCC cells [15]. Thus, the increased expression of TREM2 may be related to the fast growth, invasiveness and migration potential of gliomas.

The exact pathway that TREM2 may regulate in gliomas remains unclear. Our GSEA results indicated that TREM2 overexpression was positively correlated with KEGG apoptosis, Cromer metastasis and the KEGG chemokine pathway (Figure 6). We observed that the expression of apoptosis-related factors (cleaved caspase 3 and Bad) was lower, while the expression of anti-apoptosis (Bcl2), invasion (MMP2 and MMP9) and chemokine pathway related factors (CXCL10 and CXCR3) was higher in glioma tissues than in normal tissues. On the contrary, depletion of TREM2 significantly increased the expression of cleaved caspase 3 and Bad, while it decreased the expression of Bcl2, MMP2, MMP9, CXCL10 and CXCR3. These data suggested that these genes participate in TREM2-induced glioma progression. Several CXCL10/CXCR3 axis-related signal transduction cascades and effectors, which are important for cell survival, proliferation and apoptosis, have been determined, such as ERK, P38 and PI3K/AKT [18-20]. Interactions between chemokines and chemokine receptors were suggested to be related to the initiation and progression of cancer. A recent study demonstrates that CXCL10 and its receptor, CXCR3, are upregulated in human glioma cells [21]. CXCL10 and CXCR3 stimulate DNA synthesis and cell proliferation *in vitro*, suggesting that this chemokine could play an important role in brain tumor biology. The CXCL10/CXCR3 axis has been regarded as an important regulator in cancer cell invasion. Walser et al. reported that a small molecular weight antagonist of CXCR3 inhibits lung metastasis in a murine model of metastatic breast cancer [22]. Kawada et al. demonstrated that activation of CXCR3 with its ligands promotes colon cancer metastasis to lymph nodes [23]. In our study, TREM2 knockdown significantly suppressed the expression of CXCL10 and CXCR3, which could be interpreted as anti-proliferation and anti-invasion effects of TREM2 siRNA in glioma cells. Further investigations are required to elucidate the detailed mechanisms by which TREM2 regulates CXCL10/CXCR3 axis.

In summary, our study demonstrated that the expression of TREM2 is significantly up-regulated in glioma tissues. Depletion of TREM2 is able to suppress glioma cell growth and invasion by apoptosis, Cromer invasion and KEGG chemokine pathway. Therefore, TREM2 may be considered as an oncogene and has significant value as an unfavorable progression indicator for glioma patients, and may serve as a therapeutic target in the future.

## MATERIALS AND METHODS

### Cell culture and human glioma tissues collection

The human glioblastoma cell lines, U251, T98G, U373 and U87 cells were from the American Type Culture Collection (ATCC, Rockville, MD, USA). SHG44 cells were from the cell bank of Shanghai Biological Institute, Chinese Academy of Science (Shanghai, China). All culture media were supplemented with 10% fetal bovine serum (FBS, Hyclone, Logan, UT, USA). U87, U373 and T98G were cultured in Eagle's Minimum Essential Medium (MEM; Hyclone), while U251 and SHG44 cells were cultured in Dulbecco's modified Eagle's medium (DMEM; Hyclone). Cell lines were cultured at 37°C in a humidified atmosphere of 5% CO_2_.

A total of 70 glioma and 14 normal brain samples were obtained from Xinhua Hospital, Shanghai Jiaotong University, School of Medicine from January 2009 to December 2010. Informed consent was obtained from all patients. The patients' clinical characteristics such as age, gender, tumor size, and WHO grade, were collected for statistical analysis. The patients' prognoses were obtained from clinical services. The study protocol was approved by the local, independent ethics committee at Xinhua Hospital, Shanghai Jiaotong University School of Medicine. Written informed consent was obtained from all patients. For histological analysis, resected glioma and non-neoplastic brain tissues were fixed in formalin, embedded in paraffin and cut into 5-μm thick sections. For RT-qPCR and western blot analysis, tissues were immediately frozen in liquid nitrogen and kept at −80°C until analysis.

### Immunohistochemistry

Tissue sections were cut and mounted on slides. After de-waxing and rehydration, the sections were antigen-retrieved in 10 mm citrate buffer for 5 min at 100°C. Endogenous peroxidase activity and non-specific antigens were blocked with 3% hydrogen peroxide and serum, followed by incubation with TREM2 antibody (Sigma-Aldrich, St. Louis, MO, USA) [24] overnight at 4°C. Slides were then incubated with goat anti-rabbit secondary antibody, developed using 3,3-diaminobenzidine (DAB) solution and counterstained with hematoxylin. Immunohistochemically stained tissue sections were reviewed and scored separately by two pathologists blinded to the clinical parameters. The specimens were graded into two groups according to the extent of positivity as follows: low: < 25% of the tumor cells showed positive stain; high: >25% of tumor cells showed positive stain.

### Silencing of TREM2 by small interfering RNA

Two siRNAs targeting human TREM2 mRNA were synthesized (TREM2-siRNA1: 5′- GCCUCUUGGAAGGAGAAAUUU-3′; TREM2-siRNA2: 5′-AGAACACCUGACAACUUCUUU-3′). A non-specific scramble siRNA sequence was used as a negative control (NC: 5′- UUGUACUACACAAAAGUACUG-3′). The siRNAs were transiently transfected into U87 and U373 cells using Lipofectamine 2000 (Invitrogen, Carlsbad, CA, USA) according to the manufacturer's instruction. Assays were performed 48 h after transfection.

### RNA isolation, reverse transcription and RT-qPCR

Total RNA was extracted using TRIzol Reagent (Invitrogen) according to the manufacturer's instructions as previously described [25]. Total RNA (1 μg) was reverse-transcribed and the resulting cDNA was used as a template in real-time quantitative PCR using a standard SYBR Green PCR kit (Thermo Fisher Scientific, Rockford, IL, USA) on an ABI 7300 Real-Time PCR machine (Applied Biosystems, Foster City, CA, USA). Cycling conditions were 95°C for 10 min to activate DNA polymerase, followed by 40 cycles of 95°C for 15 s, 60°C for 45 s. Specificity of amplification products was confirmed by melting curve analysis. PCR reactions for each gene were repeated three times. Independent experiments were conducted in triplicate. GAPDH served as an internal control. The gene expression was calculated using the ΔΔ Ct method. All data represent the average of three replicates. Primer sequences were used as follows: TREM2 (NM_001271821.1), forward primer: 5′-GGAGCACAGCCATCACAGAC-3′; reverse primer: 5′-CACATGGGCATCCTCGAAGC-3′; GAPDH (NM_001256799.2), forward primer: 5′-AATCCCATCACCATCTTC-3′; reverse primer: 5′-AGGCTGTTGTCATACTTC-3′.

### Immunoblotting

Treated and untreated cells were lysed in radioimmunoprecipitation assay buffer with a freshly added protease inhibitor cocktail (Roche Applied Science, Indianapolis, IN, USA). Amounts of total protein extracts were determined using BCA assay kit (Thermo Fisher Scientific) and samples were stored at −80°C until use. Proteins were separated by sodium dodecyl sulfate-polyacrylamide gel electrophoresis (SDS-PAGE). Probing and detection of specific proteins was performed with enhanced chemiluminescence (ECL, Millipore, Bredford, USA) after antibody binding. The following antibodies were used: anti-TREM2 was from Sigma-Aldrich; antibodies against PCNA, MMP2, MMP9, CXCR3, CXCL10, Bax, Bcl-2 and cleaved caspase 3 were purchased from Abcam; anti-GAPDH was purchased from CST Biotech (Danvers, MA, USA). The immunoreactive bands were quantified by the densitometry with Image J software (NIH, USA).

### Cell proliferation assay

Cell proliferation was analyzed using the Cell Count Kit-8 (CCK-8, Dojindo Laboratories) assay. Treated and untreated cells were seeded in 96-well plates at a density of 1000-1500 cells per well and incubated for 1, 2 or 3 days. At the indicated time point, CCK8 solution was added to each well and incubated for 1 h. The absorbance value (optical density) of each well was measured at 450 nm. For each experimental condition, three wells were used. All experiments were performed thrice.

### Cell cycle distribution analysis

Treated and untreated cells were washed with ice-cold PBS once and fixed in 70% ethanol. Fixed cells were then washed in PBS and incubated with 1 mg/ml RNase A and 0.1 mg/ml propidium iodide (PI, Sigma, St. Louis, MO, USA) for 30 min at 37°C. Intensities of fluorescence signals were measured on a FACScan flow cytometer (BD Biosciences, San Jose, CA, USA). The percentage of cells in the G0/G1, S and G2/M phases was determined using FlowJo software (version 7.6.1, Tree Star, Ashland, OR, USA). All experiments were performed thrice.

### Apoptosis detected by flow cytometry

Treated and untreated cells were harvested and washed with ice-cold PBS. Annexin V and PI staining were carried out using the Annexin V-FITC Apoptosis Detection Kit (BD Biosciences, San Jose, CA, USA), according to the manufacturer's instructions. After a 20-min incubation in a dark at room temperature, the cells were immediately analyzed by FACScan flow cytometer.

### Cell adhesion assay

Treated and untreated cells were seeded onto fibronectin-coated 12-well plates at a density of 1×10^5^ cells per well and allowed to adhere at 37°C for 1 h. After non-adherent cells were washed off with PBS, attached cells were fixed in 4% paraformaldehyde and stained with GIEMSA solution. The adherent cells were photographed and counted under an Olympus inverted microscope (Lake Success, NY, USA).

### Cell migration and invasion assays

*In vitro* cell migration and invasion assays were examined using Boyden chambers containing polycarbonate filters with a pore size of 8 μm (Coring Incorporated, NY, USA). For the cell migration assay, 1 × 10^4^ cells in 100 μl MEM medium without FBS were seeded in the top chamber. In the lower chamber, 500 μl MEM with 10% FBS was added as chemoattractant. After the cells were incubated for 24 h at 37°C in a 5% CO_2_ atmosphere, cells on the top surface of the insert were removed with a cotton swab. Cells adhering to the lower surface were fixed with 4% paraformaldehyde, stained with crystal violet solution and counted under a microscope in five fields (× 200). The experiments were performed in triplicate. The procedure for the cell invasion assay was similar to the cell migration assay, except that the transwell membranes were precoated with Matrigel (BD Biosciences).

### *In vivo* tumorigenesis in nude mice

A total of 2 × 10^6^ logarithmically growing U87 cells in 0.1 ml PBS were subcutaneously injected into the right flank of 4-week-old male BALB/c nude mice (*n* = 10) (SLAC laboratory animal Center, Shanghai, China). Ten days after subcutaneous injection, the nude mice were intratumorally injected with control adenovirus (NC) or TREM2 RNAi adenovirus (TREM-RNAi) (Shanghai SBO Medical Biotechnology Company, China) every two days for 24 days. After that, the mice were killed and tumor tissues were excised and weighed. The excised tumor tissues were formalin-fixed, paraffin-embedded, sectioned and then analyzed with Ki67 immunostaining (Abcam, Cambridge, MA, USA) or TUNEL assay (Roche Applied Science, Mannheim, Germany). All animal experiments were approved by the IACUC committee at the Shanghai Jiaotong University.

### Gene set enrichment analysis (GSEA)

In this study, GBM cohort downloaded from The Cancer Genome Atlas (TCGA) was analyzed by GSEA. GSEA was performed using the GSEA software, Version 2.0.1, obtained from the Broad Institute (http://www.broad.mit.edu/gsea; ref. 20) as previously described [26-28]. Gene set permutations were performed 1,000 times for each analysis. The nominal *P* value and normalized enrichment score (NES) were used to sort the pathways enriched in each phenotype.

### Statistical analysis

GraphPad Prism (version 5.0, GraphPad Software, La Jolla, CA, USA) software was used for statistical analysis. Data are presented as the mean ± S.D. Two-tailed Student's t-test was used for comparisons between groups. Chi-square test was used to identify differences between categorical variables. The prognostic significance analysis was performed using Kaplan-Meier method and log-rank tests. Differences were considered statistically significant if *P* < 0.05.

## SUPPLEMENTARY MATERIAL FIGURE


