# Supplementary material for: Overexpression of TREM2 enhances glioma cell proliferation and invasion: a therapeutic target in human glioma
Source: Oncotarget. 2015 Oct 24;7(3):2354–66. doi: 10.18632/oncotarget.6221 (PMC4823040; doi:10.18632/oncotarget.6221)
Supplement: Supplementary file 1 [file oncotarget-07-2354-s001.pdf]

Overexpression of TREM2 enhances glioma cell proliferation and invasion: a therapeutic target in human glioma

Supplementary Material

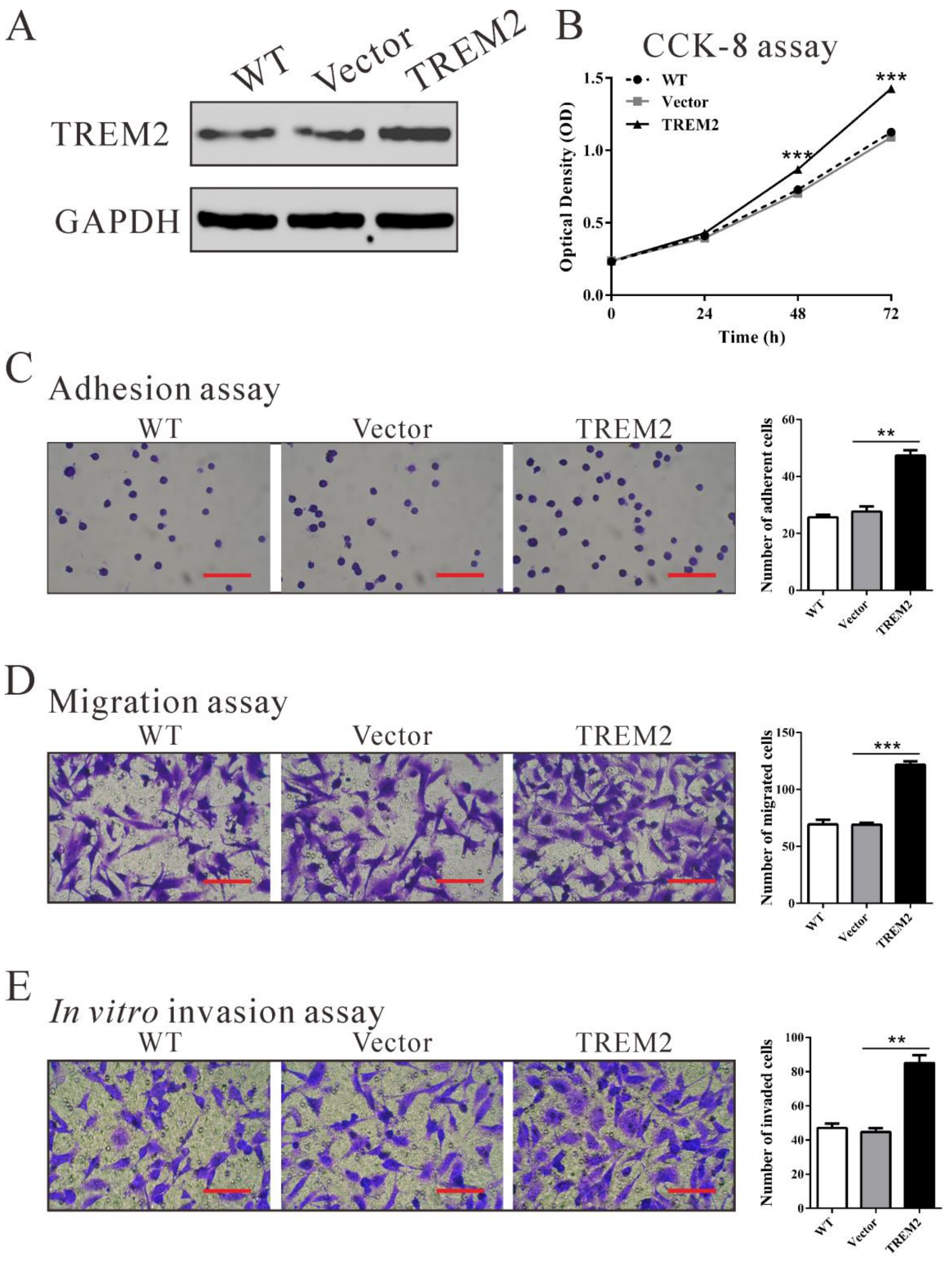

Supplementary Figure S1: Effects of TREM2 overexpression on cell proliferation, adhesion, migration and invasion of SHG44 cells. (A) Full-length TREM2 CDNA was cloned into the vector pcDNA3.1 (Invitrogen). SHG44 cells transfected with pcDNA3.1/TREM2 (TREM2) or pcDNA3.1 (Vector) by using lipofectamine 2000 (Invitrogen) and ectopic expression of TREM2 was confirmed by western blot at 48 h after transfection. TREM2 overexpression significantly promoted cell proliferation (B), cell adhesion (C), cell migration (D) and invasion (E). Scale bar: 100□m (** $P<0.01$, ***$P<0.001$ compared with Vector).
